# Conversion from Standard-Release Tacrolimus to MeltDose^®^ Tacrolimus (LCPT) Improves Renal Function after Liver Transplantation

**DOI:** 10.3390/jcm9061654

**Published:** 2020-06-01

**Authors:** Johannes von Einsiedel, Gerold Thölking, Christian Wilms, Elena Vorona, Arne Bokemeyer, Hartmut H. Schmidt, Iyad Kabar, Anna Hüsing-Kabar

**Affiliations:** 1 Department of Medicine B, Gastroenterology and Hepatology, University Hospital Münster, 48149 Münster, Germany; johannes.voneinsiedel@ukmuenster.de (J.v.E.); Christian.Wilms@ukmuenster.de (C.W.); Elena.Vorona@ukmuenster.de (E.V.); Arne.Bokemeyer@ukmuenster.de (A.B.); hepar@ukmuenster.de (H.H.S.); iyad.kabar@ukmuenster.de (I.K.); Anna.Huesing-Kabar@ukmuenster.de (A.H.-K.); 2 Department of Internal Medicine and Nephrology, University Hospital of Münster Marienhospital Steinfurt, 48565 Steinfurt, Germany

**Keywords:** MeltDose^®^, LCPT, tacrolimus, renal function, liver transplantation, C/D ratio, metabolism

## Abstract

Renal impairment is a typical side effect of tacrolimus (Tac) treatment in liver transplant (LT) recipients. One strategy to avoid renal dysfunction is to increase the concentration/dose (C/D) ratio by improving drug bioavailability. LT recipients converted from standard-release Tac to MeltDose^®^ Tac (LCPT), a novel technological formulation, were able to reduce the required Tac dose due to higher bioavailability. Hence, we hypothesize that such a conversion increases the C/D ratio, resulting in a preservation of renal function. In the intervention group, patients were switched from standard-release Tac to LCPT. Clinical data were collected for 12 months after conversion. Patients maintained on standard-release Tac were enrolled as a control group. Twelve months after conversion to LCPT, median C/D ratio had increased significantly by 50% (*p* < 0.001), with the first significant increase seen 3 months after conversion (*p* = 0.008). In contrast, C/D ratio in the control group was unchanged after 12 months (1.75 vs. 1.76; *p* = 0.847). Estimated glomerular filtration rate (eGFR) had already significantly deteriorated in the control group at 9 months (65.6 vs. 70.6 mL/min/1.73 m^2^ at study onset; *p* = 0.006). Notably, patients converted to LCPT already had significant recovery of mean eGFR 6 months after conversion (67.5 vs. 65.3 mL/min/1.73 m^2^ at study onset; *p* = 0.029). In summary, conversion of LT recipients to LCPT increased C/D ratio associated with renal function improvement.

## 1. Introduction

The calcineurin inhibitor tacrolimus (Tac) is considered a first-line immunosuppressant in liver transplant (LT) recipients [1,2,3,4]. Because of its small therapeutic window, therapy with Tac requires close drug monitoring [5]. In addition, deterioration of renal function induced by acute or chronic calcineurin inhibitor nephrotoxicity (CNIT) is a common side effect [6]. Recent studies have reported characteristics of chronic CNIT in up to 70% of LT recipients [7,8]. Furthermore, up to 8.5% of patients develop end-stage renal disease in long-term follow-up [9].

Several studies have revealed that the risk of CNIT is associated with both high Tac trough concentration and high daily Tac dose [10,11], although CNIT may occur even with low-dose regimens [12]. One potential explanation for this association is the correlation between CNIT and a fast metabolism rate of twice-daily immediate-release Tac (IR-Tac). The Tac blood concentration to daily dose ratio (C/D ratio) has been identified as a simple tool to describe patients’ metabolism rate in a steady state, in which a low C/D ratio reflects a high rate of metabolism [13,14,15]. A low IR-Tac C/D ratio is linked with higher C2 Tac blood concentrations despite comparable trough levels in patients with high C/D ratios [16]. In this regard, a low C/D ratio is strongly associated with an increased risk of CNIT and a faster decline of renal function in both kidney transplant (KT) and LT recipients [13,16,17,18,19]. Thus, increasing the C/D ratio by improving Tac bioavailability may result in better nephroprotection. One way of potentially influencing the pharmacokinetics of Tac is to change the formulation of the drug [20].

LCPT is a novel Tac formulation using MeltDose^®^ technology, in which the particle size of the drug is reduced from 10 µm to the smallest possible units (<0.1 µm), resulting in increased dissolution and thus better absorption [21]. This feature, combined with drug release over the entire intestinal tract, results in LCPT having significantly better bioavailability than other Tac formulations. Tremblay et al. showed that the intraday peak-to-trough fluctuation was approximately 30% lower for LCPT than for standard-release Tac (IR-Tac and once- daily extended-release Tac (ER-Tac)) [20]. A dose reduction of up to 30% has been observed in KT and LT recipients on LCPT [22].

Hence, we hypothesize that conversion from standard-release Tac to LCPT increases C/D ratio and thereby preserves renal function.

## 2. Materials and Methods

### 2.1. Patients and Study Design

**Figure 1:**
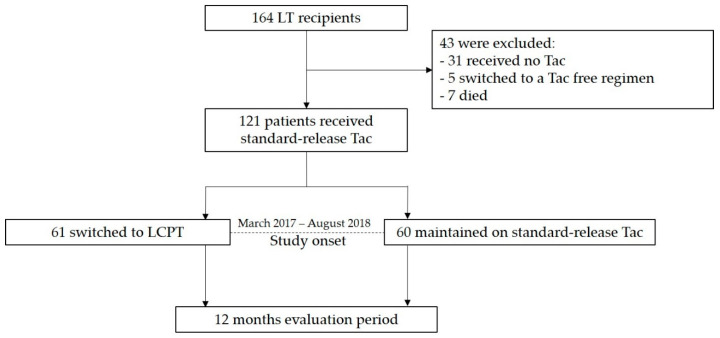
Study design and patient enrolment. A total of 164 liver transplant (LT) recipients were screened for eligibility. Only LT recipients who were started on IR- or ER-Tac (standard-release tacrolimus) and continued taking this drug until the beginning of the study were included. During the enrolment period (March 2017–August 2018), 121 patients met the inclusion criteria and were either switched to LCPT (once-daily MeltDose^®^ tacrolimus (Tac); intervention group) or maintained on standard-release tacrolimus (control group). Clinical data were analysed in a 12-month follow-up. We hypothesized that conversion from standard-release Tac to LCPT increases concentration/dose (C/D) ratio and thereby preserves renal function

Figure 1 illustrates the enrolment of the subjects in the study. This observational study was performed on patients who had undergone cadaveric liver transplantation at the University Hospital of Münster. LT recipients were included at the time of presentation at our Outpatient Transplant Clinic between March 2017 and August 2018. The study start was defined as the first appointment in this period.

At this time point, the treating physicians made a decision to either leave the patients on their usual immunosuppressive treatment (control group) or to switch them from standard-release Tac (IR- or ER-Tac) to LCPT (intervention group). Data were analysed over a 12-month follow-up. Inclusion criteria were aged over 18 years, intake of standard-release Tac before enrolment, stable graft function and an interval between transplantation and inclusion in the study of at least 1 month. LT recipients were not allowed to receive any medications or agents that could interfere with Tac. The decision about drug conversion was made by treating physicians at their own discretion.

The initial immunosuppressive regimen consisted of Tac (Prograf or Advagraf), mycophenolate mofetil (CellCept, MMF) and prednisolone (Decortin H/Soludecortin H). Tac was given at a dose of 0.1 mg/kg twice daily with a target trough concentration of 8–10 ng/mL during the first month, 6–8 ng/mL from months 2 to 3, and 3–5 ng/mL thereafter. MMF was started at a dose of 1 g twice daily and was adjusted in case of adverse effects. Initial prednisolone was given at a dose of 250 mg once daily intravenously before and immediately after LTx and was tapered stepwise. In most cases, prednisolone had been discontinued within 6–12 months after LTx.

Laboratory data were collected at study onset (at the time of conversion to LCPT or the first presentation during the above-mentioned period in the control group (t_0_)) and after 3 (t_3_), 6 (t_6_), 9 (t_9_) and 12 (t_12_) months. Serum bilirubin, alanine transaminase (ALT) and international normalized ratio (INR) were measured to assess graft function. General demographic data and information on transplantation and diagnoses were obtained from the patient records.

The C/D ratio, calculated as the ratio of Tac trough level to the corresponding daily dose, was determined 3 months before study start, at the study start and at subsequent evaluation time points. The t_0_ C/D ratio in the intervention group was determined the day before first LCPT intake. Renal function was calculated using the estimated glomerular filtration rate (eGFR) in accordance with the Chronic Kidney Disease Epidemiology Collaboration equation at the corresponding time points. The difference from baseline eGFR (t_0_) was determined at the time points of t_3_, t_6_, t_9_ and t_12_. A negative value indicates deterioration of eGFR, while a positive value indicates improvement.

The study was conducted in accordance with current medical guidelines and the Declarations of Istanbul and Helsinki. The study was also approved by the local ethics committee (Ethik Kommission der Ärztekammer Westfalen-Lippe und der Medizinischen Fakultät der Westfälischen Wilhelms-Universität Münster, No. 2016-046-f-S). Collected patient data were anonymized and written consent for collection and use of the clinical data was obtained.

### 2.2. Statistical Analysis

Statistical analysis was performed with IBM SPSS^®^ Statistics 25 for Windows (IBM Corporation, Somers, NY, USA). Normally distributed data are shown as mean ± standard deviation; non-normally distributed data are shown as median (minimum–maximum). For unrelated groups, normally distributed data were compared with a *t*-test, non-normally distributed data with the Mann–Whitney U-test and categorical variables with Fisher’s exact test. Comparison of continuous variables within a connected group was performed with the Wilcoxon signed-rank test. Pearson’s test was used to describe normally distributed data, whereas Spearman’s test was applied to non-normally distributed data. In all statistical evaluations, two-sided tests were used; a *p*-value of ≤ 0.05 was considered significant for all tests performed.

The study onset was defined as the baseline (t_0_). In the first approach, eGFR changes (t_3_-t_12_) from baseline were compared between the intervention group and the control group. In the next step, eGFR changes from every time point to baseline were compared within each group (eGFR slope). A negative value indicates deterioration in eGFR, whereas a positive value indicates an improvement in renal function.

Multivariable analysis was performed to identify independent predictors of alterations in renal function (ΔeGFR) after 12 months compared with that at baseline. For this purpose, univariable analysis with factors known to potentially influence renal function was initially performed. Variables that showed a *p*-value < 0.15 in univariable analysis were included in the multivariable analysis. Variables with a significance of <0.05 in multivariable analysis were considered significant.

## 3. Results

### 3.1. Study Population

A total of 121 patients were included in this study: 61 in the intervention group and 60 in the control group. An overview of patient characteristics, underlying diagnoses for LTx, comorbidities and immunosuppression is shown in Table 1 and Table 2. There were only small differences in the demographic data between the study groups. The control group had a more extended warm ischemic time (*p* = 0.005). As coimmunosuppression, patients received mycophenolate mofetil (MMF) at a daily dose of 1000 (500–2000) mg (LCPT) and 1500 (500–2000) mg (control), everolimus at 2.0 (0.5–5.0) mg (LCPT) and 2.0 (2.0–4.0) mg (control), prednisolone at 5.0 (5.0–7.5) mg (LCPT and control) and sirolimus at 1.0 mg (control). In the intervention group, 45 patients suffered from chronic kidney disease (CKD, categories 2–4). The control group showed a similar distribution in 39 LT recipients. No patients were in CKD category 5 or on dialysis. In the absence of kidney biopsies, the underlying renal disease remained unclear. The median interval between transplantation and study onset was 2.8 (0.1–20.8) years in the intervention group and 6.6 (0.2–16.5) years in the control group (*p* < 0.001). The reasons for a conversion from standard-release Tac to LCPT were CNIT (n = 7), neurotoxicity (n = 5) and prevention of side effects via better bioavailability of LCPT (n = 49).

### 3.2. C/D Ratio

At study start (baseline), the C/D ratio in the intervention group was comparable to that in the control group (1.68 (0.30–13.45) vs. 1.76 (0.38–7.40) ng/mL×1/mg, respectively; *p* = 0.362, Table 3). During the 12-month evaluation period, no significant changes in the C/D ratio were observed in the control group. After 12 months, the median C/D ratio was approximately at the baseline level (1.75 (0.49–6.40) ng/mL × 1/mg; *p* = 0.847). In the control group, there was a slight decrease in both the daily Tac dose at study end compared with that at baseline (2.5 (0.5–10.0) vs. 2.8 (0.5–10.0) mg, respectively; *p* = 0.084), as well as in the median Tac trough level (4.7 (1.5–14.3) ng/mL at study onset to 4.1 (1.6–15.6) ng/mL after 12 months; *p* = 0.082). However, the differences in both cases were not significant.

In contrast, the C/D ratio in patients switched to LCPT was 50% higher 12 months after conversion than that at baseline (2.52 (0.58–6.40) vs. 1.68 (0.30–13.45) ng/mL × 1/mg, respectively; *p* < 0.001). A significant increase in the C/D ratio was already observed in this group 3 months after study onset (2.03 (0.33–13.60) ng/mL × 1/mg; *p* = 0.008). Regarding the daily Tac dose, a significant reduction of 33.3% was observed after 12 months compared with that at baseline (2.0 (0.4–7.8) vs. 3.0 (1.0–22.0) mg, respectively; *p* < 0.001)). Moreover, the Tac trough level was significantly reduced at study end (4.4 (2.2–11.8) vs. 6.0 (1.5–26.9) ng/mL at study onset; *p* < 0.001).

To confirm that conditions were stable before study onset, C/D ratios, Tac doses and trough level 3 months before enrolment were also obtained. There were no significant differences between the groups at t_−3_ (Table 3) nor between study start and 3 months earlier within a group. Patients in the intervention group showed similar median C/D ratio compared with that at baseline (1.44 (0.24–6.20) vs. 1.68 (0.30–13.45) ng/mL × 1/mg, respectively; *p* = 0.204). Daily Tac dose differed significantly due to single outlier values shortly after transplant (3.0 (0.5–12.0) (t_−3_) vs. 3.0 (1.0–22.0) (t_0_) mg; *p* = 0.049), while Tac trough level showed no considerable differences (5.0 (2.4–15.3) (t_−3_) vs. 6.0 (1.5–26.9) (t_0_) ng/mL; *p* = 0.722).

No significant differences were detectable in the control group between baseline and 3 months before: C/D ratio (1.69 (0.40–9.20) vs. 1.76 (0.38–7.40) ng/mL × 1/mg, respectively; *p* = 0.626), Tac daily dose (2.5 (0.5–9.0) vs. 2.8 (0.5–10.0) mg, respectively; *p* = 0.362) and Tac trough level (4.4 (1.5–14.7) vs. 4.7 (1.5–14.3) ng/mL, respectively; *p* = 0.742).

**Figure 2:**
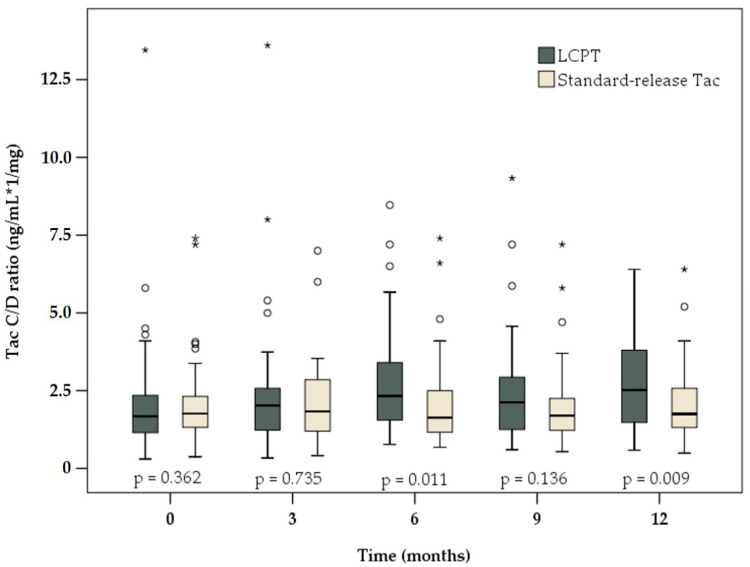
Boxplots of C/D ratio among patients receiving LCPT (dark grey) or standard-release Tac (light brown) at baseline and 3, 6, 9 and 12 months later. There were significant differences between the two study groups at 6 and 12 months after conversion. *p*-values reflect differences between the groups at each time point.

As shown in Figure 2, the C/D ratio at study end was significantly higher in patients on LCPT than in the control group (2.52 (0.58–6.40) vs. 1.75 (0.49–6.40) ng/mL × 1/mg, respectively; *p* = 0.009). The median Tac trough level and the daily dose were significantly higher in the intervention group at study onset (Table 3). After 12-month follow-up, the Tac dose in the LCPT group was significantly reduced compared with that in the control group (2.0 (0.4–7.8) vs. 2.5 (0.5–10.0) mg, respectively; *p* = 0.047). However, the Tac trough level was comparable in the two groups at study end (4.4 (2.2–11.8) vs. 4.1 (1.6–15.6) ng/mL, respectively; *p* = 0.283).

### 3.3. Renal Function

**Figure 3:**
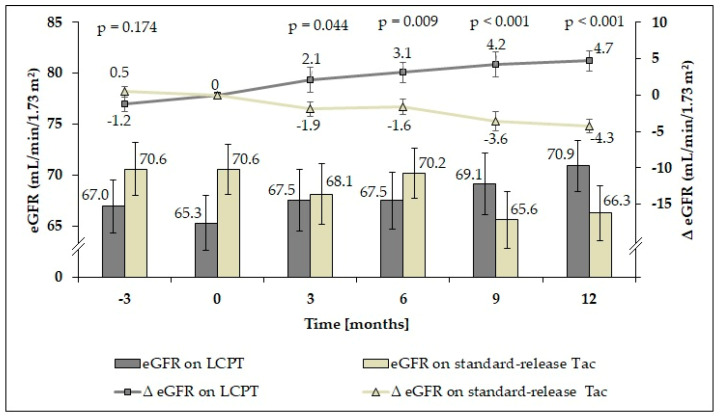
Glomerular filtration rate (eGFR; mL/min/1.73 m^2^) over time and the difference from baseline at each time point (ΔeGFR ± SEM) in each study group. Improved renal function with a significantly increased mean ΔeGFR was already observed 3 months after conversion to LCPT (dark grey). *p*-values reflect comparison of ΔeGFR between the study groups.

At baseline (study onset, t_0_), patients in the control group had a higher mean eGFR than patients switched to LCPT (Figure 3), although the difference (ΔeGFR) was not significant (*p* = 0.157). However, mean ΔeGFR in patients on LCPT had significantly improved at 6 months after conversion (*p* = 0.029). In contrast, patients on standard-release Tac showed a significant decline of mean ΔeGFR 9 months after study initiation (*p* = 0.006). Over the 12-month evaluation period, mean ΔeGFR continued to improve significantly in patients receiving LCPT (*p* = 0.001), whereas mean ΔeGFR continued to deteriorate in the control group (*p* < 0.001). In a pairwise comparison between the groups, eGFR values did not differ significantly (Supplementary Table S1).

While absolute eGFR values are meaningful to only a limited extent, eGFR slope (ΔeGFR) relative to the baseline can be used as additional empirical support (Table 4). Three months before study onset, there were no significant differences within the study groups relative to baseline. In the intervention group, renal function increased 6 months after conversion (*p* = 0.029). In contrast, LT recipients in the control group showed a significant decline of eGFR 9 months after study initiation (*p* = 0.006). Over the 12-month evaluation period, renal function continued to significantly improve in patients receiving LCPT (*p* = 0.001), whereas eGFR continued to deteriorate in the control group (*p* < 0.001).

In further analysis, the eGFR values of the patients suffering from diabetes mellitus and arterial hypertension were compared between the groups.

At every time point, patients with diabetes mellitus had significantly lower eGFR than patients without it, regardless of the study group (Table 5). However, eGFR among diabetic patients recovered in a manner similar to that of nondiabetics upon switching to LCPT. In contrast, renal function deteriorated in patients maintained on standard-release Tac in a similar fashion, regardless of diabetes.

Patients with arterial hypertension in both study groups had a lower mean eGFR than patients with normal blood pressure at each time point (Table 6). However, renal function recovered in patients treated with LCPT and deteriorated in those maintained on standard-release Tac over the course of the study, regardless of the presence of arterial hypertension.

Multivariable analysis was performed to identify independent predictors of alterations in renal function expressed as ΔeGFR (Supplementary Table S2). Conversion to LCPT was the only identified independent predictor of significant changes in eGFR.

### 3.4. Liver Function

During the entire follow-up, we monitored the graft function (Table 7). LT recipients in the LCPT group showed significantly lower serum bilirubin concentrations than the control group at all time points. However, the median values in both study groups remained within the lower part of the normal range throughout the course of the study. Regarding the parameters ALT and INR, no differences were observed between the groups.

## 4. Discussion

The present study shows that the conversion of LT recipients from standard-release Tac to LCPT was beneficial in regard to renal function. This may be due to the improved bioavailability of LCPT which led to a significant increase in C/D ratio.

Notably, the median daily Tac dose declined by 33.3% among LT recipients after conversion. A dose reduction of approximately 30% with a comparable area under the curve (AUC) was reported in recent studies of KT and LT recipients [20,24,25]. In those studies, this finding was also attributed to the greater bioavailability of LCPT.

In our cohort, the median C/D ratio among LT recipients who switched to LCPT had increased by 50% at 12 months after conversion. The C/D ratio among patients maintained on standard-release Tac remained unchanged over the 12-month period. In accordance with these data, Franco et al. described a 35% increase in the C/D ratio among KT recipients after conversion from IR-Tac and a 83.3% increase among those who were switched from ER-Tac to LCPT [26]. In the study by Rostaing et al., KT recipients had a 20% higher C/D ratio 12 months after conversion to LCPT and a 24.4% higher C/D ratio 24 months after conversion [27]. In contrast, Kamińska et al. showed that the C/D ratio of KT recipients converted from IR-Tac to ER-Tac did not change significantly [28]. To our knowledge, the present study is the first to describe a significant increase in the C/D ratio after a switch to LCPT among LT recipients.

In a previous study, we explored the impact of the C/D ratio on renal function after kidney transplantation (KTx) [14]. Fast metabolizers, defined as patients with a C/D ratio < 1.05 ng/mL × 1/mg, showed a strong association with decreased renal function compared with slow metabolizers in a 24-month follow-up. Similar results were confirmed among LT recipients in a 36-month follow-up study [13]. In that cohort, the cut-off value for fast metabolizers was defined as a C/D ratio < 1.09 ng/mL × 1/mg. In a 5-year follow-up, KT recipients with a lower Tac C/D ratio showed a higher risk of renal impairment as well as higher mortality rates [17]. Recently, several studies confirmed these findings [19,29,30] and a further negative impact of fast Tac metabolism on increased kidney allograft rejection rates and BK virus infections was demonstrated [17,18,31].

Given these results, we postulated that a higher C/D ratio after conversion to LCPT is associated with nephroprotection. Surprisingly, we already observed significant improvement of renal function 6 months after conversion. Twelve months after conversion, the mean ΔeGFR was 4.7 mL/min/1.73 m^2^ higher than at baseline. In contrast, eGFR had deteriorated significantly in patients maintained on standard-release Tac 9 months after study onset and ΔeGFR had decreased by 4.3 mL/min/1.73 m^2^ at 12 months.

After conversion to LCPT, the median trough level declined from 6.0 ng/mL at study onset to 4.6 ng/mL (month 3) without a subsequent decrease until month 12. A lower Tac trough level in the LCPT group has already been reported in a prospective study, although the same target trough level was given [27]. Alongside better bioavailability of LCPT, trough level reduction might be another reason for the increase in renal function. However, median trough levels did not vary considerably between subsequent time points (t_3_–t_12_) while renal function showed further recovery. Notably, median Tac trough levels were also slightly reduced in the control group (t_0_–t_12_), although eGFR showed further decline over the 12-month follow-up. Therefore, we postulate that improvement of bioavailability and a reduced peak Tac level after conversion to LCPT are factors more relevant to the increase in eGFR than the reduction in Tac trough levels alone.

As an explanation for the nephroprotective potential of LCPT, Schütte-Nütgen et al. hypothesized that a lower daily Tac dose results in a lower peak serum concentration (C_max_), which in turn reduces the side effects of Tac overdosing within the first hours after drug intake [17]. In a review article on LT recipients, Baraldo reported that LCPT had a similar AUC after 24 h and a similar minimal blood concentration (C_min_), but had a significantly lower C_max_ and a smaller C_max_/C_min_ fluctuation ratio when compared with IR-Tac [32]. In addition, Bunnadaprist et al. postulated that there is a reduced cumulative Tac dose in KT recipients receiving LCPT [33]. In a recent study, we also showed that fast metabolizers with a C/D ratio < 1.05 ng/mL × 1/mg had significantly higher Tac blood concentrations than slow metabolizers 2 h after Tac intake [16]. In the same study, we showed that a low C/D ratio was significantly associated with acute CNIT. Although renal biopsy is not routinely performed in LT recipients, we can assume that patients converted to LCPT suffered less frequently from CNIT. In contrast, Kamar et al. reported similar renal function in de novo KTx recipients who were randomized to LCPT or ER-Tac in a 4-week follow-up [34]. Notably, C_min_ and AUC_0–24_ were slightly higher in the LCPT group (at days 3, 7 and 14), a fact that might have influenced the results.

In the current study, the control group had an increased warm ischemic time (WIT) compared with the intervention group (~5 min). Prolonged cold and warm ischemic times can be associated with long-term allograft dysfunction [32]. Nevertheless, at the beginning of our study, the liver function parameters ALT and INR did not differ between the groups and median bilirubin was within the normal range. In a study by Laskey et al., increasing WIT during LTx was associated with a lack of renal recovery in the presence of pretransplant subacute kidney injury [35]. It was concluded that minimization of WIT could potentially avoid renal replacement therapy or the need for subsequent kidney transplantation. At the study start in our cohort, the control group showed even higher eGFR values despite increased WIT compared with the intervention group. Notably, the control group had a more extended interval between LTx and study onset than patients switched to LCPT (6.6 (0.2–16.5) vs.2.8 (0.1–20.8) years, respectively).

In regard to the Tac formulations used before study onset, IR-Tac was administered more frequently than ER-Tac in the intervention group and vice versa in the control group. A recent study on pharmacokinetics in a large transplant cohort showed similar Tac trough levels and bioavailability between these two formulations [36]. Notably, C/D ratio as well as C/D intrapatient variability was reported not to change considerably during conversion from IR-Tac to ER-Tac in KT recipients [28]. These findings justify our and others’ approach of including patients taking either one of these formulations [19,29].

In the current study, patients suffering from diabetes mellitus or arterial hypertension had reduced renal function. Interestingly, patients who were switched to LCPT (median C/D ratio increased from 1.68 to 2.52 ng/mL × 1/mg) showed considerable recovery of eGFR independent of the presence of both conditions. In accordance with these findings, Bardou et al. showed that slow Tac metabolizers (C/D ratio > 1.8 ng/mL × 1/mg) were less likely to suffer from diabetes and hypertension after LTx [37].

Finally, we recognize that our study has limitations due to its retrospective design and the limited sample size from a single-centre. In addition, in this study, we cannot provide Tac C_max,_ C_2_ (2 h after Tac intake) nor AUC, although higher C_max_ or C_2_ could potentially induce higher CNIT. Therefore, we can only hypothesize that, after conversion to LCPT, lower C_2_ was a more relevant factor to the improvement of renal function than trough level reduction. Further investigations should also include data on the concentrations of different Tac metabolites, which could be responsible for adverse effects, such as CNIT, infections and myelotoxicity [38,39]. Furthermore, given the retrospective design of this study, the study beginning in the control group had a wide range from March 2017 until August 2018 and the time period from LTx to the beginning of the study was significantly increased compared with that in the intervention group. The longer Tac exposure in the control group might have had a negative influence on renal function in this cohort. However, at t_0_, the control group showed even higher eGFR values than patients converted to LCPT (70.6 ± 19.3 vs. 65.3 ± 21.1, respectively).

Another limitation of the study is that the reasons for conversion to LCPT in our study were taken only from the clinical reports from our Outpatient Transplant Clinic. In addition, in contrast to the case for KTx recipients, renal biopsy is not routinely performed in LT recipients which limits our ability to analyse CNIT before study onset.

## 5. Conclusions

To the best of our knowledge, this is the first study to show that conversion from standard-release Tac to LCPT increases the C/D ratio in LT recipients associated with renal recovery. This finding was independent of known risk factors for renal impairment. Prospective studies are needed to confirm our findings.

## Figures and Tables

**Table 1:** Patient characteristics.

	LCPT (n = 61)	Standard-Release Tac (n = 60)	*p*-Value
Age at LTx (years)	46.3 ± 16.7	48.8 ± 12.4	0.348 ^a^
Age at study onset (years)	51.0 ± 15.9	56.1 ± 12.7	0.054 ^a^
Height (m)	1.72 ± 0.087	1.73 ± 0.094	0.714 ^a^
Weight (kg)	79.4 ± 20.8	76.7 ± 16.5	0.420 ^a^
BMI (kg/m^2^)	26.7 ± 6.1	25.6 ± 5.0	0.310 ^a^
Sex (male/female)	29 (47.5%) / 32 (52.5%)	38 (63.3%) / 22 (36.7%)	0.101 ^b^
CIT (h)	11.3 ± 2.6	10.5 ± 2.4	0.065 ^a^
WIT (min)	38.9 ± 9.2	43.8 ± 8.7	0.005 ^a^
Number of grafts			0.255 ^b^
One	56 (91.8%)	49 (81.7%)	
Two	4 (6.6%)	8 (13.3%)	
Three	1 (1.6%)	3 (5.0%)	
Blood type			0.545 ^b^
A	28 (47.5%)	30 (50.0%)	
B	6 (10.2%)	6 (10.0%)	
AB	6 (10.2%)	2 (3.3%)	
O	19 (32.2%)	22 (36.7%)	
Hepatitis B antigen (positive)	5 (8.2%)	7 (11.9%)	0.556 ^b^
Hepatitis C antibody (positive)	9 (14.8%)	8 (13.6%)	1.000 ^b^
Recipient CMV IgG (positive)	34 (57.6%)	27 (45.0%)	0.201 ^b^
Donor CMV IgG (positive)	32 (56.1%)	37 (63.8%)	0.450 ^b^

Statistics: Values shown as mean ± standard deviation or number (percentage). ^a^ *t*-test, ^b^ Fisher’s exact test. LCPT, once-daily MeltDose^®^ tacrolimus; Tac, tacrolimus; LTx, liver transplantation; BMI, body mass index; CIT, cold ischemic time; WIT, warm ischemic time; CMV, cytomegalovirus.

**Table 2:** Underlying diagnoses for LTx, comorbidities and immunosuppression at study start.

	LCPT (n = 61)	Standard-Release Tac (n = 60)	*p*-Value
Principal diagnosis			0.455
Alcoholism	9 (14.8%)	16 (26.7%)	
Viral hepatitis	15 (24.6%)	15 (25.0%)	
Genetically related metabolic disease	7 (11.5%)	5 (8.3%)	
Toxic: nutritional or NASH	3 (4.9%)	1 (1.7%)	
Autoimmune liver disease	11 (18.0%)	13 (21.7%)	
Other	16 (26.2%)	10 (16.7%)	
Arterial hypertension	36 (59.0%)	37 (61.7%)	0.853
Diabetes mellitus	18 (29.5%)	17 (28.3%)	1.000
Hyperlipidaemia	19 (31.1%)	14 (23.3%)	0.415
CKD at study start			0.598
CKD 2	18 (29.5%)	21 (35.0%)	
CKD 3a	16 (26.2%)	10 (16.7%)	
CKD 3b	9 (14.8%)	7 (11.7%)	
CKD 4	2 (3.3%)	1 (1.7%)	
Tac formulation at study onset			<0.001
Immediate-release Tac	43 (70.5%)	22 (36.7%)	
Extended-release Tac	18 (29.5%)	38 (63.3%)	
Co-immunosuppression			0.060
MMF	34 (55.7%)	35 (58.3%)	
Everolimus	13 (21.3%)	5 (8.3%)	
Prednisolone	3 (4.9%)	10 (16.7%)	
Sirolimus	0	1 (1.7%)	
None	11 (18.0%)	9 (15.0%)	
Reasons for a switch to LCPT			
CNIT	7		
Neurotoxicity	5		
Preventions of side effects	49		

Statistics: Values shown as number (percentage). All *p*-values from Fisher’s exact tests. LCPT, once-daily MeltDose^®^ tacrolimus; Tac, tacrolimus; LTx, liver transplantation; NASH, nonalcoholic steatohepatitis; CKD, chronic kidney disease (categories set with reference to [23]). MMF, mycophenolate mofetil; CNIT, calcineurin inhibitor nephrotoxicity.

**Table 3:** Tacrolimus concentration/dose (C/D) ratio, daily dose and blood trough concentration.

	LCPT	Standard-Release Tac	*p*-Value
Tac C/D ratio (ng/mL × 1/mg)			
3 months before (n = 54 vs. 58)	1.44 (0.24–6.20) on s-r-Tac	1.69 (0.40–9.20)	0.344
At study onset (n = 61 vs. 60)	1.68 (0.30–13.45) on s-r-Tac	1.76 (0.38–7.40)	0.362
After 3 months (n = 61 vs. 60)	2.03 (0.33–13.60)	1.83 (0.41–7.00)	0.735
After 6 months (n = 61 vs. 60)	2.33 (0.77–8.47)	1.63 (0.68–7.40)	0.011
After 9 months (n = 61 vs. 60)	2.13 (0.60–9.33)	1.70 (0.54–7.20)	0.136
After 12 months (n = 61 vs. 60)	2.52 (0.58–6.40)	1.75 (0.49–6.40)	0.009
Tac daily dose (mg)			
3 months before (n = 54 vs. 58)	3.0 (0.5–12.0) on s-r-Tac	2.5 (0.5–9.0)	0.056
At study onset (n = 61 vs. 60)	3.0 (1.0–22.0) on s-r-Tac	2.8 (0.5–10.0)	0.044
After 3 months (n = 61 vs. 60)	2.0 (0.8–8.0)	2.5 (0.5–9.0)	0.330
After 6 months (n = 61 vs. 60)	2.0 (0.8–5.0)	2.5 (0.5–7.0)	0.248
After 9 months (n = 61 vs. 60)	2.0 (0.4–6.0)	2.5 (0.5–9.0)	0.060
After 12 months (n = 61 vs. 60)	2.0 (0.4–7.8)	2.5 (0.5–10.0)	0.047
Tac trough level (ng/mL)			
3 months before (n = 54 vs. 58)	5.0 (2.4–15.3) on s-r-Tac	4.4 (1.5–14.7)	0.087
At study onset (n = 61 vs. 60)	6.0 (1.5–26.9) on s-r-Tac	4.7 (1.5–14.3)	0.005
After 3 months (n = 61 vs. 60)	4.6 (0.5–13.1)	4.4 (2.2–10.4)	0.863
After 6 months (n = 61 vs. 60)	4.7 (1.5–12.7)	4.1 (2.0–10.9)	0.022
After 9 months (n = 61 vs. 60)	4.3 (1.5–15.1)	4.0 (1.9–10.1)	0.867
After 12 months (n = 61 vs. 60)	4.4 (2.2–11.8)	4.1 (1.6–15.6)	0.283

To confirm that conditions were stable before enrolment, values 3 months prior to study onset are given for all patients who had already undergone liver transplantation (n = 54 vs. 58). In the intervention group (LCPT), values 3 months before and the day before the first LCPT intake (study onset) were determined when s-r-Tac was administered. LCPT, once-daily MeltDose^®^ tacrolimus; Tac, tacrolimus; s-r-Tac, standard-release tacrolimus. *p*-values from Mann–Whitney U-test.

**Table 4:** Slope analysis (ΔeGFR) of glomerular filtration rate (eGFR; mL/min/1.73 m^2^).

Time Point	Estimate	95% Confidence Limit		*p*-Value
		Lower	Upper	
LCPT				
−3 months vs. baseline	−1.2	−3.2	0.8	0.223
3 months vs. baseline	2.1	−1.3	5.5	0.219
6 months vs. baseline	3.1	0.3	6.0	0.029
9 months vs. baseline	4.2	0.8	7.6	0.015
12 months vs. baseline	4.7	1.9	7.5	0.001
Standard-release Tac				
−3 months vs. baseline	0.5	−1.0	1.9	0.547
3 months vs. baseline	−1.9	−3.9	0.0	0.053
6 months vs. baseline	−1.6	−3.8	0.6	0.154
9 months vs. baseline	−3.6	−6.1	−1.1	0.006
12 months vs. baseline	−4.3	−6.2	−2.3	<0.001

The “estimate” value describes the difference between the respective time point and the baseline (ΔeGFR). A negative value shows a decline and a positive value an improvement of eGFR. LCPT, once-daily MeltDose^®^ tacrolimus; Tac, tacrolimus; *p*-values within a group are relative to the baseline.

**Table 5:** Glomerular filtration rate (eGFR; mL/min/1.73 m^2^) in diabetic and nondiabetic patients.

Time Point	LCPT			Standard-Release Tac		
	Diabetics (n = 18)	Non-Diabetics (n = 43)	*p*-Value	Diabetics (n = 17)	Non-Diabetics (n = 43)	*p*-Value
t_0_	52.6 ± 21.3	70.3 ± 19.0	0.003	56.0 ± 20.3	76.3 ± 15.6	<0.001
t_3_	56.2 ± 21.2	72.3 ± 20.0	0.013	51.2 ± 17.6	74.9 ± 18.1	<0.001
t_6_	56.3 ± 19.2	73.0 ± 18.4	0.004	56.5 ± 20.3	75.5 ± 15.1	<0.001
t_9_	57.1 ± 22.1	74.4 ± 17.7	0.007	53.2 ± 22.2	71.5 ± 16.5	0.002
t_12_	56.3 ± 19.5	77.2 ± 13.4	<0.001	49.9 ± 22.5	72.7 ± 16.6	<0.001

LCPT, once-daily MeltDose^®^ tacrolimus; Tac, tacrolimus; t_0_ to t_12_, time points (months). eGFR values shown as mean ± standard deviation. *p*-values from *t*-test.

**Table 6:** Glomerular filtration rate (eGFR; mL/min/1.73 m^2^) in patients with and without arterial hypertension.

Time Point	LCPT			Standard-Release Tac		
	Arterial Hypertension (n = 36)	Normal Blood Pressure (n = 25)	*p*-Value	Arterial Hypertension (n = 37)	Normal Blood Pressure (n = 23)	*p*-Value
t_0_	59.3 ± 20.7	74.3 ± 18.7	0.006	66.8 ± 21.0	76.6 ± 14.5	0.056
t_3_	63.4 ± 22.4	73.1 ± 19.2	0.111	64.6 ± 22.4	74.2 ± 16.7	0.121
t_6_	62.6 ± 20.1	74.2 ± 18.5	0.038	65.5 ± 20.4	77.6 ± 12.7	0.015
t_9_	65.1 ± 19.9	74.7 ± 20.7	0.120	63.2 ± 21.8	70.4 ± 16.3	0.222
t_12_	66.7 ± 17.0	76.9 ± 18.3	0.042	61.2 ± 22.4	74.5 ± 15.6	0.015

LCPT, once-daily MeltDose^®^ tacrolimus; Tac, tacrolimus; t_0_ to t_12_, time points (months). eGFR values shown as mean ± standard deviation. *p*-values from *t*-test.

**Table 7:** Assessment of liver function over time in each study group.

	LCPT (n = 61)	Standard-Release Tac (n = 60)	*p*-Value
Bilirubin (mg/dL)			
At study onset	0.4 (0.2–1.3)	0.6 (0.2–2.0)	0.001
After 3 months	0.4 (0.2–2.2)	0.5 (0.2–4.0)	0.006
After 6 months	0.4 (0.2–1.2)	0.6 (0.2–2.1)	0.010
After 9 months	0.4 (0.2–1.2)	0.5 (0.2–1.9)	0.001
After 12 months	0.5 (0.2–1.2)	0.6 (0.2–2.7)	0.011
ALT (U/L)			
At study onset	20 (8–102)	21 (9–117)	0.431
After 3 months	24 (8–78)	22 (10–140)	0.348
After 6 months	20 (6–92)	18 (8–448)	0.406
After 9 months	20 (7–202)	20 (7–138)	0.997
After 12 months	20 (9–104)	20 (7–380)	0.696
INR			
At study onset	1.0 (0.9–2.2)	1.0 (0.9–1.6)	0.765
After 3 months	1.0 (0.9–2.3)	1.0 (0.9–1.3)	0.871
After 6 months	1.0 (0.9–1.3)	1.0 (0.9–1.6)	0.969
After 9 months	1.0 (0.9–1.3)	1.0 (0.9–1.5)	0.634
After 12 months	1.0 (0.9–1.3)	1.0 (0.9–1.5)	0.217

LCPT, once-daily MeltDose^®^ tacrolimus; Tac, tacrolimus; ALT, alanine transaminase; INR, international normalized ratio; *p*-values from Mann–Whitney U-test.

## Supplementary Materials

The following are available online at https://www.mdpi.com/2077-0383/9/6/1654/s1, Table S1: Glomerular filtration rate (eGFR; mL/min/1.73 m^2^) over time. Table S2: Univariate and multivariate analysis of variance on eGFR at 12 months relative to baseline (study onset; t_0_).
