# Supplementary material for: Conversion from Standard-Release Tacrolimus to MeltDose® Tacrolimus (LCPT) Improves Renal Function after Liver Transplantation
Source: J Clin Med. 2020 Jun 1;9(6):1654. doi: 10.3390/jcm9061654 (PMC7356524; doi:10.3390/jcm9061654)
Supplement: Supplementary file 1 [file jcm-09-01654-s001.pdf]

Conversion from Standard-Release Tacrolimus to MeltDose® Tacrolimus (LCPT) Improves Renal Function after Liver Transplantation

Johannes von Einsiedel, Gerold Thölking, Christian Wilms, Elena Vorona, Arne Bokemeyer, Hartmut H. Schmidt, Iyad Kabar and Anna Hüsing-Kabar

Table S1. Glomerular filtration rate (eGFR; mL/min/1.73 m^2) over time.

Time point	LCPT mean ± SD	Standard-release Tac mean ± SD	*p*-value
t_{-3}	66.9 ± 19.0	70.6 ± 19.6	0.321
t_0	65.3 ± 21.1	70.6 ± 19.3	0.157
t_3	67.5 ± 21.4	68.1 ± 20.8	0.886
t_6	67.5 ± 20.1	70.2 ± 18.7	0.479
t_9	69.1 ± 20.6	65.6 ± 20.2	0.399
t_{12}	70.9 ± 18.1	66.3 ± 21.0	0.216

LCPT, once-daily MeltDose® tacrolimus; t_{-3} to t_{12}, evaluation time point (months); SD, standard deviation. *p*-values from t-test.

Table S2. Univariate and multivariate analysis of variance on eGFR at 12 months relative to baseline (study onset; t_0).

	Univariate analysis				Multivariate analysis			
	Estimate	95% CI lower	95% CI upper	*p*-value	Estimate	95% CI lower	95% CI upper	*p*-value
Sex (male vs. female)	−2.72	−6.40	0.95	0.145	−1.00	−6.98	4.98	0.437
Age at study onset (per year)	−0.06	−0.19	0.06	0.325	N/A	N/A	N/A	N/A
Study group (LCPT vs. standard-release Tac)	9.00	5.71	12.26	< 0.001	9.00	0.78	17.23	<0.001
Tac formulation at study onset (IR- vs. ER-Tac)	3.37	−0.25	7.00	0.068	2.13	−5.75	10.00	0.801
Time between LTx and study onset (months)	−0.02	−0.05	0.01	0.263	N/A	N/A	N/A	N/A
Arterial hypertension (yes vs. no)	0.36	−3.40	4.11	0.852	N/A	N/A	N/A	N/A
Diabetes (yes vs. no)	−2.02	−6.05	2.00	0.322	N/A	N/A	N/A	N/A
WIT (minutes)	−0.04	−0.25	0.17	0.695	N/A	N/A	N/A	N/A

Regression coefficient estimates as well as lower and upper 95% confidence limits (95% CI) regarding eGFR 12 months after study onset relative to baseline (t_0). A negative estimated value for continuous variables describes a decrease per unit and for categorial variables, that the first category has a deteriorated eGFR. LCPT, once-daily MeltDose® tacrolimus; IR-Tac, immediate-release tacrolimus; ER-Tac, extended-release tacrolimus; LTx, liver transplantation; WIT, warm ischemic time.
